# Sunscreen Posts on Twitter in the United States and Canada, 2019: Content Analysis

**DOI:** 10.2196/29723

**Published:** 2021-07-19

**Authors:** Sajjad S Fazel, Emma K Quinn, Chelsea A Ford-Sahibzada, Steven Szarka, Cheryl E Peters

**Affiliations:** 1 Department of Oncology Cumming School of Medicine University of Calgary Calgary, AB Canada; 2 CAREX Canada Simon Fraser University Vancouver, BC Canada; 3 Cancer Epidemiology and Prevention Research Cancer Care Alberta Alberta Health Services Calgary, AB Canada; 4 School of Health Sciences University of Northern British Columbia Prince George, BC Canada

**Keywords:** sunscreen, skin cancer, Twitter, misinformation, prevention, skin, social media, health promotion, melanoma

Skin cancer is a growing burden in Canada and the United States. One effective prevention method is the use of sunscreen; however, low sunscreen use [1] coupled with the spread of misinformation online can hinder health promotion activities.

Health-related social media posts (including sunscreen) may shape risk-related behaviors of users, so it is important to understand the accuracy of such posts [2].

Twitter’s Application Program Interface was used to search for tweets in English containing the word “sunscreen” posted in Canada and the United States (May 1 to August 31, 2019). We used thematic content analysis to elicit the accuracy, sentiment, and theme of the tweets.

Tweets containing verifiable information (that could be assessed as factual or not) were analyzed for accuracy and coded as either “accurate” or “inaccurate” based on current evidence. All tweets were coded for sentiment (positive or negative).

Themes were analyzed using an a priori list of codes based on our previous study [3] and inductively modified based on emergent themes. Differences were tested using the chi-square statistic or the Fisher exact test.

**Figure 1:**
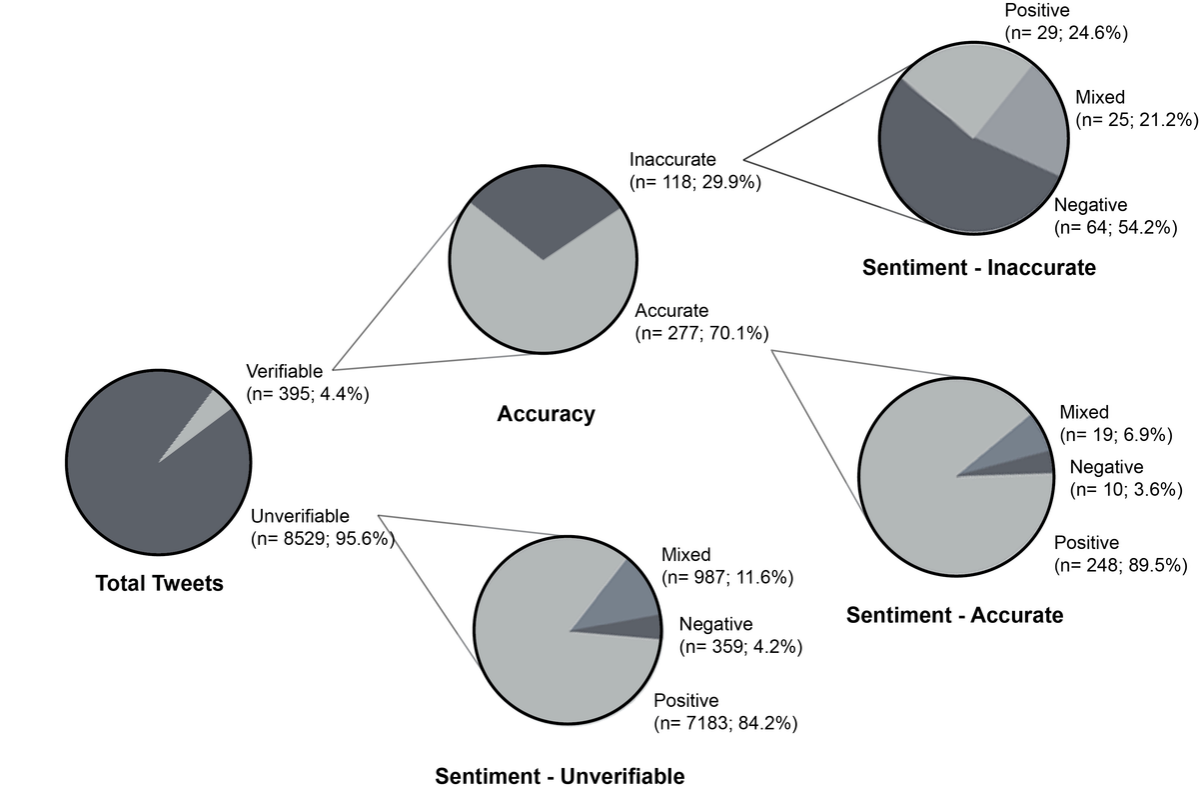
Comparison of sentiments between accurate, inaccurate, and unverifiable sunscreen tweets originating from Canada and United States between May 1, 2019 and August 31, 2019.

In total, 9176 tweets were collected; 167 retweets and 85 irrelevant tweets were excluded. The remaining 8924 tweets were analyzed for accuracy (where applicable), sentiment, and theme. The observed percentage agreement between the coders for sentiment and accuracy was 76%. Only 395 tweets (4% of the total) contained verifiable information and were analyzed for accuracy. Among these, 277 (70%) were accurate and 118 (30%) were inaccurate (Figure 1).

The most common themes were personal story (n=5425, 61%), tips and recommendations (n=2591, 28%), and advertisements (n=457, 5%). The top theme for accurate and inaccurate tweets was tips and recommendations (n=171, 56%) and personal story (n=90, 62%), respectively.

The sentiment analysis found that 7460 (84%) of tweets had a positive sentiment, 1031 (11%) were mixed or neutral, and 433 (5%) were negative. Among the accurate tweets, the majority had a positive sentiment toward sunscreen (n=248, 89%), while over half (n=64, 54%) of the inaccurate tweets had a negative sentiment. Interestingly, inaccurate tweets were more likely to have any engagement than accurate tweets (Table 1).

We found that most tweets were personal stories and not verifiable for accuracy. This suggests that misinformation about sunscreen may not be an important contributor to low sunscreen use, as also noted by Silva et al [4]. The sentiment analysis found that over 80% of all sunscreen tweets had a positive sentiment toward sunscreen use, which is similar to our previous study on sunscreen information in traditional media sources [3].

This study was limited to Twitter; further research on sunscreen misinformation using other social media platforms is recommended.

In conclusion, sunscreen misinformation was limited, but misinformation was more likely to have engagement from users. Organizations may have better success in promoting sunscreen use by producing tailored, engaging sunscreen and cancer prevention messages [5]. Furthermore, it may be beneficial for physicians and health organizations to share messages using lived experience, which may increase reach and engagement online.

**Table 1:** Comparison of Twitter data between verifiable and unverifiable tweets: Canada and United States, 2019.

Category and subcategory		Verifiable tweets		*P* value (accurate vs inaccurate tweets)	Unverifiable tweets, n (%)	*P* value (all verifiable vs unverifiable tweets)
		Accurate tweets, n (%)	Inaccurate tweets, n (%)			
**Sentiment**				<.001		<.001
	Positive	248 (89)	29 (25)		7183 (84)	
	Mixed	19 (7)	25 (21)		987 (12)	
	Negative	10 (4)	64 (54)		359 (4)	
**Engagement^a^**				.04		.68
	0	96 (35)	29 (25)		2689 (32)	
	1-5	126 (46)	66 (56)		4269 (50)	
	6-10	18 (18)	13 (11)		711 (8)	
	>10	37 (13)	10 (8)		860 (10)	
**Followers^b^**				.049		.61
	0-200	73 (26)	28 (24)		2011 (24)	
	201-500	55 (20)	38 (32)		2222 (26)	
	501-1000	58 (21)	24 (20)		1675 (20)	
	>1000	91 (33)	28 (24)		2621 (31)	
**Attached URL**				<.001		<.001
	Yes	205 (74)	60 (51)		4349 (51)	
	No	72 (26)	50 (49)		4180 (49)	
**Type of URL**				.30		<.001
	Social media	199 (88)	50 (83)		4214 (97)	
	News	5 (2)	0 (0)		88 (2)	
	Health organizations	4 (2)	1 (2)		18 (0.4)	
	Peer-reviewed journal websites	1 (0.5)	1 (2)		2 (0.05)	
	Other	17 (8)	8 (13)		268 (6)	
**Attached media**				.02		.86
	Yes	57 (21)	13 (11)		1482 (17)	
	No	220 (79)	105 (89)		7047 (83)	
**Type of media**				.87		.26
	Photo	46 (81)	11 (85)		1095 (74)	
	Video	1 (2)	0 (0)		82 (5)	
	Animated GIF	10 (17)	2 (15)		305 (21)	

^a^Engagement was defined as the total number of “likes,” “retweets,” “quote tweets,” and “replies” for each tweet.

^b^Followers was defined as the number of individual Twitter accounts following the user.
